# Misinformation and Facts about Breast Cancer Screening

**DOI:** 10.3390/curroncol29080445

**Published:** 2022-08-09

**Authors:** Daniel B. Kopans

**Affiliations:** Harvard Medical School, Boston, MA 02114, USA; dkopans@verizon.net; Tel.: +1-617-584-4584

**Keywords:** breast, cancer, screening

## Abstract

Quality medical practice is based on science and evidence. For over a half-century, the efficacy of breast cancer screening has been challenged, particularly for women aged 40–49. As each false claim has been raised, it has been addressed and refuted based on science and evidence. Nevertheless, misinformation continues to be promoted, resulting in confusion for women and their physicians. Early detection has been proven to save lives for women aged 40–74 in randomized controlled trials of mammography screening. Observational studies, failure analyses, and incidence of death studies have provided evidence that there is a major benefit when screening is introduced to the general population. In large part due to screening, there has been an over 40% decline in deaths from breast cancer since 1990. Nevertheless, misinformation about screening continues to be promoted, adding to the confusion. Despite claims to the contrary, a careful reading of the guidelines issued by major groups such as the U.S. Preventive Services Task Force and the American College of Physicians shows that they all agree that most lives are saved by screening starting at the age of 40. There is no scientific support for using the age of 50 as a threshold for screening. All women should be provided with the facts and not false information about breast cancer screening so that they can make “informed decisions” for themselves about whether to participate.

## 1. Introduction

The deaths from the COVID pandemic that could have been avoided have emphasized the tragic consequences resulting from the promulgation of inaccurate information and ignoring science. Unfortunately, “alternative facts” have been generated about breast cancer screening that go back decades. Confusion has resulted from the misinformation that has been published due to poor peer review in some of the most prestigious journals [1,2,3,4]. These erroneous analyses are then reported to the public by the media, which is unable to understand some of the complexities of the claims being made, resulting in confusing messages. The following reviews just a few of the many false issues that have been raised over the years that are not supported by science. These have been used in an effort to reduce access to screening and to distract from the scientific evidence that supports the fact that annual screening starting at the age of 40 saves the most lives.

Early detection has secondary benefits such as a reduced need for mastectomies, less need for axillary dissection with the attendant reduced risk of lymphedema, and less toxic systemic therapy [5,6,7], but the following discussion will concentrate on the main benefit, which is mortality reduction and the fact that randomized controlled trials have proven that early detection saves lives for women aged 40–74.

## 2. The Decades-Long Effort to Reduce Access to Breast Cancer Screening

I suspect that most are unaware of the fact that there has been an almost continuous effort, dating back to the 1950s, to limit access to breast cancer screening. This is probably, and primarily, an effort to save money, but opponents know that if they told women they did not want to pay to save their lives, there would be “a discussion” that they would lose. Consequently, numerous scientifically unsupportable claims have been made to limit access.

As long ago as the 1950s, it was claimed that breast cancer was systemic before it could be found so that earlier detection would have no advantage. This was the origin of the effort to develop systemic treatments.

In the 1960s, based on the standardization of the mammographic technique, the first randomized controlled trial (RCT) of screening was conducted by the Health Insurance Plan of New York (HIP). HIP proved that lives could be saved by earlier detection [8]. Since HIP, the importance of early detection has been reinforced by multiple RCTs of breast cancer screening [9].

In the 1970s, the Breast Cancer Detection Demonstration Project (BCDDP)) was conducted to challenge the claim that it was not possible to screen large numbers of women efficiently and effectively. More than 275,000 women had annual mammography and clinical breast examinations over a 5-year period. In the BCDDP, 40% of the cancers were found only by mammography [10], proving the feasibility of population-based early detection.

In the mid-1970s, while the BCDDP was underway, it was claimed that the low doses of radiation needed for mammography might cause more cancers than would be cured [11]. This “radiation scare” resulted in the BCDDP stopping screening for women aged 40–49. It is now known that radiation risk to the breast is primarily for teenage women and those in their early twenties, likely related to incomplete terminal differentiation of the lobules. This risk falls rapidly with increasing age so that by the time women are in their 40s, there is no measurable risk. It is impossible to prove that there is “no risk,” but even extrapolating the risk from younger women, it is far below even the smallest benefit [12,13]. Based on the evidence, even those groups that are trying to reduce access to screening have stopped raising radiation risk as a major concern.

The debate continued as to what age screening should begin. In 1989, major medical groups, including the U.S. National Cancer Institute (NCI), reached a “consensus” and advised that women aged 40–49 be screened every 1 to 2 years and women aged 50 and over be screened annually [14]. However, there were those at the NCI who did not support screening, particularly for women in their forties, so that in 1993, with a change in leadership, the NCI ignored the science and reversed its initial advice by deciding that women should wait until the age of 50 and be screened every 2 years [15].

Statistical power is critical for scientific validity. If a trial does not include sufficient numbers of women, there may be a reduction in deaths, but it will be ignored since it does not reach “statistical significance.” In 1993, as a supposedly science-based organization, the NCI used an inappropriate statistical approach by analyzing the data for women aged 40–49 separately from older women in randomized controlled trials. They ignored the fact that the trials had not been planned to evaluate age subgroups and were not designed with sufficient power to permit legitimate “subgroup” analyses. As we showed, and the NCI ignored, it was impossible for the trials to show a significant mortality reduction of the expected 25% within 5 years of the start of the trials that the NCI was requiring [16]. Based on an unplanned retrospective subgroup analysis of trials lacking statistical power for this analysis to be legitimate and, for the first time in history, ignoring the advice of their main advisory group known as the National Cancer Advisory Board (NCAB), the NCI dropped support for screening women aged 40–49 and advised women aged 50 and over to be screened every 2 years despite having no data to support reducing the interval between screens from one year to two [17].

Due to concerns raised about the NCI decision in 1993, and under new leadership, the NCI agreed, in 1997, to a consensus development conference (CDC) to examine the value of screening women in their forties [18]. Despite reassurances that the CDC would be a neutral review and would be conducted without NCI influence (it was the NCI’s guidelines that were under review), the CDC was organized by a declared opponent of screening working at the NCI and a review panel with several members having a conflict of interest (undisclosed NCI funding) was convened. The CDC was provided with a longer follow-up of the RCTs that showed an unambiguous, statistically significant decline in breast cancer deaths for women aged 40–49 even when analyzed separately [19]. Despite the fact that the CDC had been organized to evaluate these latest data, they were ignored (not even mentioned), and the CDC falsely claimed that there was insufficient support for screening women in their forties [20] and that the misinformation was spread by the media [21]. The updated information was subsequently reviewed by the NCAB, and recognizing that they provided scientific proof and based on NCAB advice, the NCI once again supported screening starting at the age of 40 [22]. Soon after, the NCI decided it would no longer issue guidelines.

In 2007, the American College of Physicians (ACP), having supported screening, suddenly changed course and advised women to wait until the age of 50 and be screened every 2 years [23]. The ACP and the United States Preventive Services Task Force (USPSTF) are closely allied, and the USPSTF followed suit with the same recommendations in 2009 [24].

In 2015, the American Cancer Society, a previously staunch supporter of annual mammography starting at the age of 40, submitted to political pressure and developed very strange recommendations. They initially stated that “women should have the opportunity to begin annual screening between the ages of 40 and 44 years (qualified recommendation),” but went on to recommend a scientifically unjustified hybrid recommendation stating that women might want to delay screening until the age of 45 and be screened annually until the age of 55 and then biennially after that [25].

In 2016, the USPSTF reaffirmed its advice to delay screening [26].

All three groups (USPSTF, ACP, and ACS) agree that most lives are saved by screening starting at the age of 40 [25,26,27], but in 2019, the ACP reaffirmed their support for the USPSTF and advised women that they should wait until the age of 50 and be screened every 2 years [28].

The USPSTF and the ACP advised delaying participation because of the “harms” of screening, which they claimed were “false positives” (a misnomer for women recalled for a few extra pictures and an ultrasound); “overdiagnosis” of cancers that would never harm a woman in her lifetime and, if left alone, would regress and disappear; and “overtreatment,” which is the unnecessary treatment of “overdiagnosed” cancers.

Of course, if it even occurs, “overdiagnosis” is the fault of pathologists and not screening, and oncologists, not screening, are responsible for deciding treatment. Blaming screening for these is analogous to blaming the engines in our cars for traffic accidents. Regardless, the claims of “overdiagnosis” are based on the incredibly rare cases of clinically evident cancer that have disappeared without treatment. These are so rare that they can be considered true “miracles.” In fact, the relative handful that has been reported has all been clinically evident. No one has ever observed a mammographically detected invasive breast cancer disappear on its own [29]. Since this never happens, it is misleading to advise women that they should delay screening until age 50 to reduce “overdiagnosis” since these cancers, if they even exist, will still be detected at age 50. Delaying screening will not avoid “overdiagnosed” cancers, but women will die unnecessarily if screening is delayed.

Unfortunately, although the USPSTF, ACS, and ACP all agree that delaying screening will result in avoidable deaths, they stress their claims of “harms.” They have not directly stated to women and their physicians the actual number of lives that will be lost. They have not told women that, if their guidelines are followed, it is predicted that thousands will die unnecessarily that could be saved by annual screening starting at the age of 40.

The groups that advise waiting until age 50 (or 45 as per the ACS) and then screening every 2 years instead of annually have not made it clear that the only “harm” that is affected by delaying screening is the “false positive rate.” The term “false positive” is a misleading, pejorative choice of words. Women are not being told (falsely) that they have breast cancer. Instead, based on the findings of their screening study, these women are simply being asked to return for a few extra pictures and sometimes an ultrasound to be careful, and, contrary to the “false positive” terminology used, most are reassured that there is no evidence of cancer.

Approximately 2% of the women screened are advised to have a very safe, image-guided needle biopsy using local anesthesia in an outpatient setting, and 20–40% of these are found to have breast cancer.

## 3. How Frequently Should Women Be Advised to Be Screened?

Despite the fact that there has never been an RCT to evaluate the optimum time interval between screens (annual vs. biennial or longer), there is a large amount of inferential data [30,31,32] supporting annual screening. Not surprisingly, the shorter the time between screens, the greater the likely benefit [33].

The NCI supports CISNET (the Cancer Intervention and Surveillance and Modeling Network), which includes six groups that have developed separate computer models to predict the result of interventions. All six agree that the most lives are saved by annual screening starting at the age of 40 [34]. The CISNET models show that if the ACS, ACP, or USPSTF guidelines are followed, thousands of lives will be lost that could be saved by annual screening starting at the age of 40 [35].

## 4. The RCTs Proved That Screening Saves Lives for Women Aged 40–74

RCTs are the only way to eliminate biases such as “lead time,” “length bias,” “selection bias,” etc., which can make it difficult to evaluate the benefit of an intervention such as screening for breast cancers. The RCTs of screening proved that early detection reduces deaths among women aged 40–74, which are the ages of the women who participated in the trials [36]. This does not mean that women under age 40 and over age 74 may not also benefit from screening, but we have clear scientific proof for women aged 40–74 [19].

It is important to understand that RCTs underestimate the benefit. Once a woman is allocated to a study arm or a control arm, whatever happens to her is attributed to that assignment. Some of the women who are invited to be screened refuse the invitation. This is termed “noncompliance.” In order to avoid selection bias, if they should die from breast cancer, they are still counted as a death among the screened women and dilute the benefit from screening. If a woman is assigned to a control arm, she is not prevented from obtaining a mammogram on her own outside the trial (called “contamination”). If her life is saved by the mammogram, she is still counted as an unscreened control. Since there was “noncompliance” and “contamination” in all of the trials, the RCTs likely underestimated the benefit. Although the RCTs proved that screening reduces deaths, because of noncompliance and contamination, they do not provide an accurate measure of the absolute mortality reduction. Observational studies suggest that if all women participate in screening, deaths may be reduced by over 50%.

## 5. There Is No Scientific Support for Using the Age of 50 as a Threshold for Screening

This cannot be stated too emphatically. There is no scientific support for using the age of 50 as a threshold for screening. It originated because investigators in the HIP trial were interested in identifying whether menopause had any influence on their results. Since they had not collected any menopausal data on their participants, they chose the age of 50 as a surrogate for menopause and, retrospectively, evaluated women aged 40–49 separately from those aged 50–64. Ignoring the fact that this subgroup analysis of the younger women lacked statistical power, they misinterpreted their results and claimed that screening was more robust for women aged 50–64 because there appeared to be an immediate decrease in deaths (likely statistical fluctuation with early small numbers), while the decline in deaths among the younger women did not begin to appear for 5–7 years. In fact, periodic screening is unlikely to produce an immediate reduction in deaths, while the “delayed benefit” is exactly what would have been expected [37]. Based on that faulty analysis, it was falsely claimed that screening was more robust for older women, and the age of 50 continues to be falsely claimed as a legitimate threshold for starting screening.

Not only was the HIP analysis not scientifically supported, but, in fact, there are no data that have not been grouped and averaged that show that any of the parameters of screening change abruptly at menopause, age 50, or any other age [38]. False claims of a sudden change at age 50 [39] have arisen by taking factors such as breast cancer detection and deaths and by grouping ages together and then taking the average of women aged 40–49 compared with the average for women aged 50–74, rather than examining rates by individual age. This takes a variable that actually changes steadily with increasing age and makes it appear to change suddenly when there is no such sudden change [40]. The only starting age for screening based on science and evidence is the age of 40.

## 6. The Benefit of Screening, Proven by RCTs, Is Confirmed by Observational Studies in the General Population

RCTs have proven that screening and early detection reduce deaths. As noted above, differences in death rates between study and control groups prove the benefit, but because of noncompliance and contamination, their results are diluted and do not provide absolute measures. The reduction in deaths has been confirmed when screening is introduced into the general population, in which women who have access to screening have much better survival than those who do not [34,41,42,43,44,45,46,47,48,49,50,51,52,53,54,55]. In these observational studies, it has been found that women aged 40 and over who actually participate in screening have a greater than 40% reduction in deaths.

## 7. Failure Analyses Add More Support for Screening

Another way to evaluate the benefit of early detection is using “failure analysis.” What is different about women who die from breast cancer than those who do not? In a study of women who died from breast cancer in the Harvard teaching hospitals, 71% of the deaths were among the 20% of women who were not participating in screening despite all the women having access to modern therapy [56]. In an analysis by Spencer et al., the results were similar [57]. Among women who die from breast cancer, despite access to modern therapy, most deaths were among the smaller percentage of women who had not participated in screening.

## 8. “Incidence of Death” Is Another Way to Evaluate the Effects of Screening

A very large study of more than 500,000 women in Sweden provides additional evidence of the benefit of screening. The risk of dying from breast cancer was reduced by 41% within 10 years of diagnosis for women who participated in screening compared with those who did not [58].

## 9. Data from the U.S. Strongly Support the Benefits of Screening

It has never been explained why, in the U.S., our National Cancer Institute’s Surveillance Epidemiology and End Results (SEER) National database has never included the method by which breast cancers are detected (MOD). This has led to numerous claims opposing mammography screening that cannot be challenged using SEER data. Unfortunately, I suspect that this is not accidental. History shows that the NCI has not been a supporter of screening, particularly for women aged 40–49, so it may well be that failing to collect data on MOD has been a conscious decision. Thus, in the U.S., we have no direct data on the results of mammography screening.

Nevertheless, by examining the data that have been collected (incidence numbers), it can be estimated that screening began in the mid-1980s in large enough numbers to influence national statistics. At this time, there was a relatively sudden increase in breast cancer incidence [59] that likely signaled the beginning of screening at a population level with sufficient numbers to be seen in national incidence estimates. Since there is no nationally organized screening program in the U.S., screening did not begin suddenly for all women. Perhaps 20% of women had at least one mammogram in the mid-1980s. It appears that participation in screening gradually increased in the 1980s and 1990s and then plateaued at the end of the 1990s when it is estimated that approximately 70% of women had had at least one mammogram [60]. I would speculate that these data support a prolonged “prevalence peak” as more and more women began to participate in screening in the late 1980s and 1990s. Prevalence screening likely ended by 1999 and explains why there was a fairly abrupt decline in “incidence” that began in 1999.

The participation in screening in the mid-1980s suggested by the data is likely the reason for the sudden decline in deaths from breast cancer that began in 1990. Data from the Connecticut Tumor Registry [61] dating back to the 1940s (SEER only began in 1974) show that the death rate from breast cancer had been unchanged for decades. As the rate of local breast cancers increased fairly abruptly and the relative rate of advanced cancers began to fall, the death rate from breast cancer began to fall in 1990, 5–7 years after the start of screening, as has been the case in the RCTs. As more and more women have participated in screening and cancer detection has improved, the death rate has continued to decline. A recent review of the SEER data shows that there are now more than 40% fewer women dying each year from breast cancer, saving an estimated 600,000 lives since 1990 [59]. There is no question that therapy has improved. Lives are being prolonged, but there is still no cure for metastatic breast cancers. Curing breast cancer is only possible when it is treated earlier. We do not know why 40,000 women still die each year despite advances in treatment because SEER does not collect MOD. What we can say is that they were not cured by therapy. The failure analyses noted earlier suggest that many of those who die are likely not participating in screening.

## 10. Men with Breast Cancer Have Worse Outcomes than Women

There are not many other ways to evaluate the benefit of screening. One is to compare deaths from breast cancer among men to deaths among women. The death rate from breast cancer for women has fallen dramatically since 1990 and continues to fall. Over the same time period, deaths among men with breast cancer actually increased for several years and then fell back to 1990 levels and have stayed at that level [62]. Treatment for breast cancer in men is similar to treatment for women. Men, however, generally present with more advanced cancers than women. This would suggest that the differences in deaths are likely due to the fact that women are being screened and men are not.

There is no doubt that therapy for breast cancer has improved, but treating these cancers earlier saves the most lives.

## 11. Most Recently, Fundamental Errors in the Canadian National Breast Screening Studies Have Been Confirmed

The Canadian National Breast Screening Studies (CNBSS) have been major outliers among the RCTs of breast cancer screening. Unlike other trials, they failed to show any benefit from mammography and clinical breast examination screening for women aged 40–49 and no benefit from mammography screening for women aged 50–59. The CNBSS results have been used to reduce access to screening for women in their 40s in Canada and around the world.

The only way to “prove” that medical intervention is efficacious is an RCT. RCTs are designed to produce identical groups. In an RCT of screening, if conducted properly, the same number of women in both groups will develop breast cancer, and the same number of women will die from breast cancer if nothing else is done. If one of the groups is offered screening and the other is not, and statistically, significantly fewer women die in the screening arm compared with the controls, then this is proof that screening saves lives.

In order for the groups to be identical, it is critical that the participants be divided randomly. Say I performed an RCT for treating breast cancer in which I chose to test an obsolete chemotherapeutic agent (similar to the outdated mammography used in the Canadian National Breast Screening Studies) to determine whether it was superior to no treatment at all, but I first examined all the women who volunteered for the trial, allowing me to identify the women with advanced cancer prior to assigning them to the treatment or control arm. Then I assigned them on open lists so that I could, undetectably, assign women to whichever arm I wanted out of random order, and I placed more women with advanced cancers in the treatment arm than the control arm, and it turned out that there were more deaths among the treated women than among controls in the early years of the trial, and, at the end of the trial, there was no difference in survival between both groups, and I concluded that there was no benefit from ANY form of systemic therapy. You would, legitimately, wonder how my trial passed a human studies review since I had violated the main “rules” for RCTs. I would likely be cited for ethics violations, and you would ask how I was able to publish the results from my flawed trial, and you would be correct in arguing that my publications should be withdrawn.

This would, in fact, be the correct response to such a compromised trial, yet similar violations of the rules for RCTs took place in the Canadian National Breast Screening Studies (CNBSS). For inexplicable reasons, these trials passed institutional reviews, peer reviews in journals, and subsequent reviews by various other panels. Instead of being ignored as having unreliable results, these trials have been praised by supposed trial experts [63], and their negative results have been used for decades to deny women access to screening [64].

In other RCTs of screening, a general population was first identified, and then, without knowing anything about the women, they were randomly assigned to the study or the control arms. The women allocated to the study arms were invited to participate in screening. The women allocated to the control arms had their “usual care.” The CNBSS was different. Volunteers were first recruited. This means that there was a likelihood that women “self-selected.” Perhaps women who were more health conscious agreed to participate. They tend to have better outcomes. Regardless, they were likely not representative of a general population, so the results would not be “generalizable,” yet they have been applied to all women.

In order to be certain that assignment to study or control arms is random, “blinded allocation” is required. Nothing can be known about the participants prior to allocation that could be used to inadvertently or intentionally “load” one side or the other. This fundamental rule was violated in the CNBSS. The investigators have admitted, and an independent review has verified, that most of the women in the CNBSS underwent a clinical breast examination (CBE) by highly trained nurses [65] prior to allocation. These CBEs identified women with suspicious clinical findings before they were assigned to the study or control arm. Of course, you might ask why these women, many of whom had clinically evident cancers and could not benefit from mammography screening, were not excluded from a trial testing the value of mammography screening.

The preallocation CBE was a major violation, but this was compounded by the fact that the CBE results were provided to the coordinators, who determined to which group the women would be assigned. Had this still been a blinded assignment, then it is likely that the women with signs or symptoms of breast cancer would have been assigned equally to both arms, and their participation would have only diluted the benefit. However, the CNBSS violated another basic rule. Instead of blinded assignment, the women were assigned on open lists. The coordinators knew which lists would result in mammography screening and could assign women, out of random order, to either group in a process that could not be traced.

A problem was first recognized [66] when the trialists reported 19 women with advanced cancers allocated to the screening arm while only 5 were assigned to the control arm in CNBSS1. This proved to be a “statistically significant” difference [67]. The trialists have falsely argued that this was because “mammography finds more of everything.” They ignored the fact that 17 of the 19 advanced cancers were evident on the preallocation CBE.

Numerous other published facts have indicated that assignments were not all random and that the screening arm was “loaded,” but the unsupportable denials by the investigators have always been accepted by various reviewers. No one has ever suggested that the imbalances were intended by the trialists, but the trials’ designs and executions made imbalances possible. The coordinators were not experienced in RCTs and may well have, naively, wanted to be certain that a woman with probable cancer had a mammogram and assigned her out of random order to be certain that she had a mammogram.

It seemed that the facts would never be known. I personally wrote to MacMahon and Bailar, who were brought in to review the trials [68]. I cited the obvious need to interview the coordinators (with protection from any retribution) [69] to determine whether they had assigned women out of random order as the data suggested, but the CNBSS investigators would never permit them to be interviewed to find out what actually took place.

In March of 2021, I presented a talk virtually to the Toronto Society of Breast Imaging, in which I outlined the concerns raised by the published data about the CNBSS and, in particular, the indications of nonrandom allocation. Soon after, I received an email from an attendee who had been an X-ray technologist in the CNBSS. She attested to the fact that she had witnessed nonrandom allocation of women with clinical evidence of breast cancer who were assigned out of random order to the mammography arms [70]. An extensive effort to interview any remaining workers in the CNBSS has confirmed the fact that not only were women with signs or symptoms assigned out of random order but that, in fact, many were actually recruited into these trials of screening despite the fact that they could not benefit from screening [71,72,73].

You would also think that trials of mammography screening would use state-of-the-art systems. What has also been ignored over the years is the well-documented fact that the CNBSS used some inferior, obsolete mammography systems. The technologists had no special training in obtaining mammograms and used obsolete positioning that did not image the axillary tail of the breast where many cancers develop. Grids to reduce scatter X-rays were not employed, likely causing some small cancers to be obscured. Their own reference physicist cited problems with their imaging [74]. I was one of three radiologists whom the investigators chose to conduct a blinded review of their mammograms [75]. This review confirmed the poor quality of the images. It showed that for much of the studies, the images were poor to unacceptable [76]. Evidence of the poor quality of the images is suggested by the fact that in the CNBSS, fewer cancers were detected by mammography alone (30%) than with older techniques used 10 years earlier in the BCDDP (40%). These and other problems also worked against the demonstration of the benefit of mammography. Most reviews of these trials that have been undertaken over the years have excluded experts in mammography screening. It is likely that the inexpert reviewers have made the false assumption that “a mammogram is a mammogram.”

There are numerous reasons to withdraw the results from these trials. Assigning women with more advanced cancers in nonrandom order to the screening arms, imaging using obsolete systems by technologists who had no training, and radiologists who had minimal if any training in interpreting mammograms—the CNBSS results were clearly imbalanced against screening. It is not surprising that these trials are major outliers among the other RCTs by not showing a benefit of mammography for women at any age from 40–59.

The data have long shown, and an eyewitness has now verified, that there were fundamental flaws in the execution of the CNBSS, rendering their results unreliable. They should not be used to advise women on screening guidelines, and the publication of their results should be withdrawn.

## 12. Conclusions

The most rigorous medical studies have proven that mammography screening reduces deaths from breast cancer for women aged 40–74. This has been confirmed by observational studies, failure analyses, and incidence of death studies. Mammography is far from perfect. It does not find all cancers, and even earlier detection does not guarantee a cure. Therapy has improved, but there is still no cure for advanced cancers, and screening has helped cut the death rate from breast cancer in half. Computer models all show that annual screening saves the most lives. The age of 50 has no scientific support as a threshold for screening.

The promulgation of misinformation needs to stop. All women and their physicians need to be provided with scientifically valid information to make “informed decisions.” Women should be advised that annual screening starting at the age of 40 saves the most lives.

## Data Availability

Not applicable.
